# Ischemic Postconditioning and Subanesthetic S(+)-Ketamine Infusion: Effects on Renal Function and Histology in Rats

**DOI:** 10.1155/2015/864902

**Published:** 2015-08-27

**Authors:** Marco A. C. de Resende, Alberto V. Pantoja, Bruno M. Barcellos, Eduardo P. Reis, Thays D. Consolo, Renata P. Módolo, Maria A. C. Domingues, Alexandra R. Assad, Ismar L. Cavalcanti, Yara M. M. Castiglia, Norma S. P. Módolo

**Affiliations:** ^1^Pós-Graduação em Anestesiologia, Faculdade de Medicina de Botucatu, Universidade Estadual Paulista (UNESP), 18618-970 Botucatu, SP, Brazil; ^2^Serviço de Anestesiologia, Departamento de Cirurgia, Universidade Federal Fluminense (UFF), 24033-900 Niterói, RJ, Brazil; ^3^Faculdade de Medicina de Botucatu, UNESP, 18618-970 Botucatu, SP, Brazil; ^4^Departamento de Patologia, UNESP, 18618-970 Botucatu, SP, Brazil; ^5^Departamento de Anestesiologia, UNESP, 18618-970 Botucatu, SP, Brazil

## Abstract

*Background*. Ischemic postconditioning (IP) in renal Ischemia reperfusion injury (IRI) models improves renal function after IRI. Ketamine affords significant benefits against IRI-induced acute kidney injury (AKI). The present study investigated the effects of IP and IP associated with subanesthetic S(+)-ketamine in ischemia-reperfusion-induced AKI. *Methods*. Forty-one Wistar rats were randomized into four groups: CG (10), control; KG (10), S(+)-ketamine infusion; IPG (10), IP; and KIPG (11), S(+)-ketamine infusion + IP. All rats underwent right nephrectomy. IRI and IP were induced only in IPG and KIPG by left kidney arterial occlusion for 30 min followed by reperfusion for 24 h. Complete reperfusion was preceded by three cycles of 2 min of reocclusion followed by 2 min of reperfusion. Renal function was assessed by measuring serum neutrophil gelatinase-associated lipocalin (NGAL), creatinine, and blood urea nitrogen (BUN). Tubular damage was evaluated by renal histology. *Results*. Creatinine and BUN were significantly increased. Severe tubular injury was only observed in the groups with IRI (IPG and KIPG), whereas no injury was observed in CG or KG. No significant differences were detected between IPG and KIPG. *Conclusions*. No synergic effect of the use of subanesthetic S(+)-ketamine and IP on AKI was observed in this rat model.

## 1. Introduction

Ischemia-reperfusion injury (IRI) is an inevitable phenomenon during transplantation [1]. IRI is also one of the causes of delayed graft function and acute kidney injury (AKI) after transplantation and is associated with multiple processes, including inflammatory cascade activation, ion accumulation, free reactive oxygen species (ROS) formation, endothelial dysfunction, platelet aggregation with microembolization, and immune activation [2].

AKI incidence continues to increase despite the use of new biomarkers in clinical care to anticipate diagnosis and improve treatment [3]. Reducing IRI by creating resistance against IRI through preconditioning and postconditioning the organ has become an area of increasing interest. The principle of brief alternating cycles of arterial occlusion and reperfusion prior to complete reperfusion after a prolonged ischemic episode, so-called “ischemic postconditioning” (IP), in kidneys was recently reviewed for several animal models by van den Akker et al., demonstrating benefits with respect to organ damage and kidney function [1].

Cell injury induced by reactive oxygen species (ROS) is a determinant of IRI. Ketamine infusion appears to inhibit lipid peroxidation and the amount of ROS [4]. The addition of low-dose ketamine prior to induction [5] or continuous infusion of the S(+) isomer diminishes the responses of proinflammatory cytokines during and after cardiac surgery involving cardiopulmonary bypass [6]. Recently, evidence of the involvement of NMDA (N-methyl-D-aspartate) in AKI has been reported [7]. It has been demonstrated that AKI is associated with both the activation of NMDA receptors and significant oxidative stress. The antagonism of various allosteric sites of NMDA receptors, including the polyamine-binding site inhibitor ketamine, affords significant benefit against IR-induced AKI. Additionally, at low doses and with continuous infusion, the noncompetitive NMDA antagonist S(+)-ketamine could minimize sympathomimetic action and present anti-inflammatory action.

The aim of this study was to investigate the effects of IP and IP associated with subanesthetic S(+)-ketamine on renal function and histology in rats.

## 2. Materials and Methods

### 2.1. Experimental Protocol

**Figure 1:**
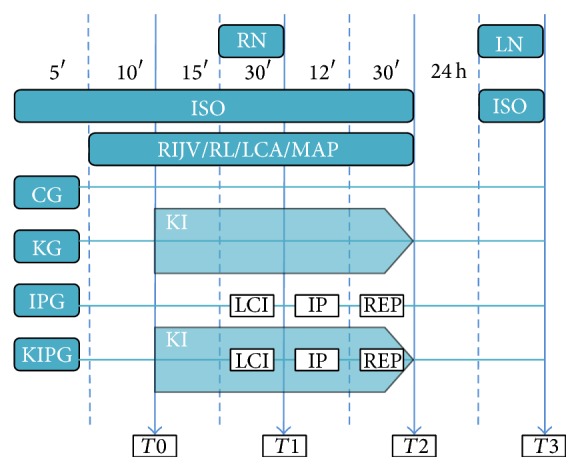
Experimental algorithm. ISO: isoflurane; RIJV: right internal jugular vein cannulation; RL: ringer lactate; LCA: left carotid artery; MAP: mean arterial pressure; RN; right kidney nephrectomy; LN: left kidney nephrectomy; KI: S(+)-ketamine infusion; LCI: left artery clamping; IP: ischemia-reperfusion cycles (12 min total); REP: full reperfusion; CG: control group; KG: S(+)-ketamine group; IPG: ischemic postconditioning group; KIPG: S(+)-ketamine ischemic postconditioning group.

The rats received care that complied with the Guide for the Care and Use of Laboratory Animals published by the National Institutes of Health and with Brazilian law regarding animal experimentation. Following approval from the Research Ethics Committee for Animal Experimentation (protocol number 946/2012) of Botucatu Medical School, São Paulo State University (UNESP), 41 male Wistar rats weighing 300–500 g were allocated into four groups: CG, control group, *n* = 10; KG, subanesthetic S(+)-ketamine continuous infusion, *n* = 10; IPG, IP, *n* = 10; and KIPG, subanesthetic S(+)-ketamine continuous infusion + IP, *n* = 11. All rats were submitted to right nephrectomy. Postconditioned rats (IPG and KIPG) were also submitted to left kidney arterial occlusion for 30 minutes (min); complete reperfusion for 24 hours (h) was preceded by three cycles, 2 min of reperfusion followed by 2 min of reocclusion each, for a total of 12 min. To achieve anesthesia inhalation, the rats were housed in a bell jar suitable for small animals. Anesthesia was initiated with 4% isoflurane (vaporizer, Ohmeda) with a total flow of 1 L·min^−1^ oxygen and 1 L·min^−1^ air. Once the rats could be manipulated, the isoflurane concentration was reduced to 2% and was administered by a nonrebreathing mask system under spontaneous respiration (Figure 1).

The temperature of the rats was maintained between 35.5°C and 37.5°C using a thermal blanket and was monitored with a rectal thermometer. The right internal jugular vein (RIJV) was dissected and cannulated with a 24 GA venocath for infusion of Ringer lactate solution (RL) (3 mL·kg^−1^·h^−1^) in all groups and subanesthetic (1.25 mg·kg^−1^·h^−1^) S(+)-ketamine continuous infusion only in KG and KIPG, in compliance with the Food and Drug Administration (FDA) publication Guidance for Industry: Estimating the Maximum Safe Starting Dose in Initial Clinical Trials for Therapeutics in Adult Healthy Volunteers [8]. The left carotid artery (LCA) was dissected and cannulated with a 24 GA venocath to monitor invasive arterial pressure through the transducer of a Datex-Engström recorder (Finland) and to obtain blood samples. In KG and KIPG, S(+)-ketamine drug infusion was initiated for 15 min before laparotomy and maintained until the end of the experiments. The following parameters were recorded: mean arterial pressure and rectal temperature at 15 min after the beginning of the anesthesia or after the cannulation of the RIJV and LCA (*T*0), after 30 min of left renal artery clamping (*T*1), and after 30 min of reperfusion and the cycles of IP (*T*2). The following biochemical variables were evaluated: serum neutrophil gelatinase-associated lipocalin (NGAL), creatinine, sodium, and blood urea nitrogen (BUN) at *T*0, *T*2, and after 24 h of reperfusion (*T*3). Each blood sample (2 mL) was accompanied by bolus infusion of 4 mL of Ringer lactate. Laparotomy was performed under cutaneous infiltration with 0.7 mL of 0.125% bupivacaine. To prevent compensation by the nonischemic kidney, right nephrectomy was performed in all groups, as proposed in Dobashi's modified method [9]. For arterial clamping, a temporary atraumatic Schwartz clip (Rs-5452, Roboz Surgical Instrument Co. Inc., Gaithersburg, MD, USA) was used. Following closure of the abdominal wall, the rat was awakened, taken to the animal center, and placed in an individual cage in a temperature-controlled environment with free access to food and water. Twenty-four hours later, the rats were submitted to laparotomy under anesthesia using the same technique. Left nephrectomy was performed, and the third arterial blood sample (*T*3) was collected directly from the heart. The rats were then immediately sacrificed by intracardiac injection of bupivacaine.

### 2.2. Renal Function Analysis

Serum NGAL (Rat NGAL ELISA Kit, Bioporto Diagnostics, Gentofte, Denmark), creatinine, and BUN (standard kits) were estimated to evaluate renal function. Serum sodium was used to evaluate hemodilution among the groups.

### 2.3. Histological Analysis

The left kidneys were processed for histological analysis. Once removed, the kidneys were sectioned longitudinally and stored in separate vials. They were maintained in Duboscq-Brazil solution for the first 48 h and were then preserved in 70% ethanol. Histological sections were stained with hematoxylin-eosin. Histological analysis was performed by a single pathologist blinded to the origin of the groups studied. The scale described by Park et al. [10] was used to determine the histological severity of tubular cell injury, which is graded from 1 to 5 as follows: grade 0, no lesions; grade 1, mild injury, less than 10% tubular cell necrosis; grade 2, mild to moderate injury, 10 to 25%; grade 3, moderate to severe injury, 25 to 50%; grade 4, severe injury, 50 to 75%; and grade 5, severe to very severe injury, greater than 75% tubular cell necrosis.

### 2.4. Statistical Analysis

All data are expressed as the means ± standard deviations (SDs). Repeated measures analysis of variance (ANOVA) and the contrast test were used to assess the behavior of the biochemical and monitored variables for three periods within each experimental group. One-way repeated measures ANOVA was used to determine whether the evolution of the variables was different among the groups (interaction effect). In these analyses, logarithmic transformation (natural log) was applied to the data due to the lack of normality, according to the Kolmogorov-Smirnov test. The nonparametric Kruskal-Wallis ANOVA was used to compare body weight among the four groups. The nonparametric histological scores were analyzed between IPG and KIPG using the Mann-Whitney test. In all instances, values of *p* < 0.05 were considered statistically significant. The statistical analysis was processed using SAS System version 6.11 for Windows (SAS Institute, Inc., Cary, NC, USA).

## 3. Results

The body weight values were similar for all groups (*p* = 0.43). One-way repeated measures ANOVA of the monitoring variables (mean arterial pressure and temperature) showed no significant effects due to the interaction (*p* = 0.18 and *p* = 0.30, resp.). No functional impairment or histological injury was detected in CG and KG. Contrast analysis of repeated measures ANOVA of the biochemical variables showed that the values obtained at *T*0 (basal) were significantly lower than at *T*2 (with 30 min of reperfusion, after the cycles of IP) and *T*3 (after 24 h) in CG and KG, and the increase in values was progressive and significant between *T*0, *T*2, and *T*3 for IPG and KIPG (Table 1). No significant differences were detected between *T*2 and *T*3 values for CG and KG.

### 3.1. Effects of S(+)-Ketamine on Renal Morphology and Function

A subanesthetic dose of S(+)-ketamine alone did not alter the functional parameters evaluated or promote any detectable injury in the histological analysis (grade 0, Park et al.). Repeated measures ANOVA showed a significant variation for sodium only in KG. Despite performing nephrectomy, ketamine infusion did not worsen the evolution of the biochemical variables.

### 3.2. Effects of Postconditioning on Renal Morphology and Function

IP alone (PG) promoted progressive increases in all renal function parameters (creatinine, NGAL, and BUN), which exhibited statistically significant differences from the basal values (*T*0). Longitudinal contrast analysis of the variables within the group showed that *T*0 < *T*2 < *T*3. However, one-way repeated measures ANOVA, which verifies whether the progression in values is different between the groups, showed interaction effects for creatinine (*p* < 0.0001) and BUN (*p* < 0.0001). Contrast analysis between the groups verified that creatinine and BUN were very similar in IPG and KIPG (Table 2). All PG rats showed some degree of histological lesion, according to Park et al.

### 3.3. Effects of S(+)-Ketamine and Postconditioning on Renal Morphology and Function

The values of renal function parameters obtained for KIPG were greater than those obtained for IPG; however, longitudinal contrast analysis showed similar progressions in these values between these groups. Repeated measures ANOVA (time effect) for KIPG showed statistically significant values for creatinine, NGAL, and BUN. One-way repeated measures ANOVA showed that only creatinine and BUN were statistically significant regarding the interaction effect; contrast analysis confirmed that IPG and KIPG showed very similar progressions in values. As observed for the IPG rats, all KIPG rats demonstrated tubular injury during the histological analysis.

### 3.4. Histopathology

**Figure 2:**
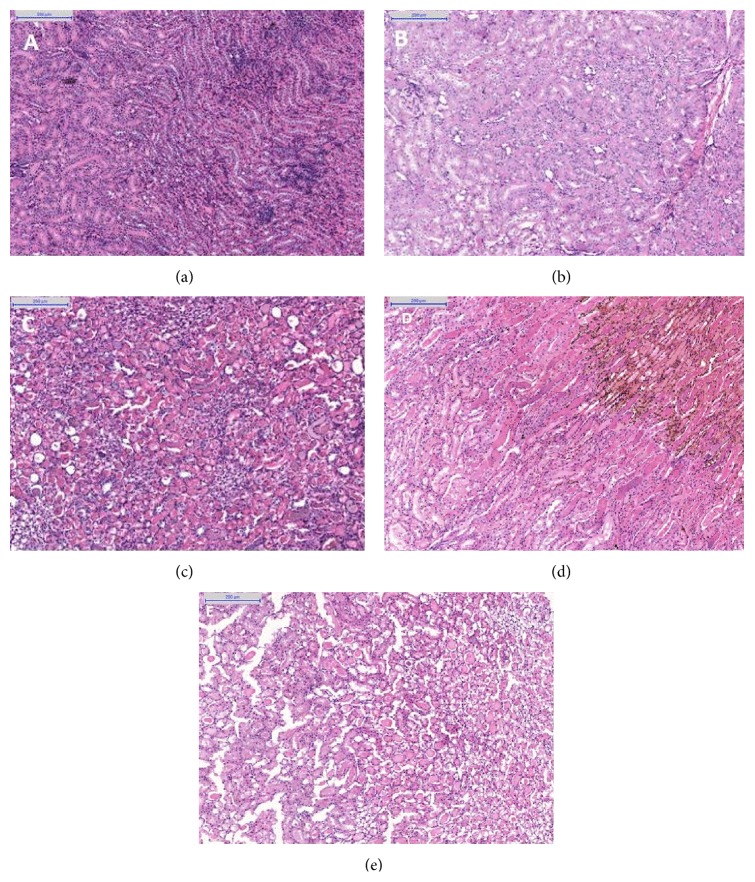
Representative light micrographs of rat kidneys, magnification 200x. Hematoxylin-eosin stain of kidney sections, graded for severity of tubular injury, according to Park et al. [10] (a) CG, left kidney, grade 0 = no lesions; (b) KG, left kidney at 24 h, grade 0; (c) IPG, left kidney at 24 h, grade 4 (severe injury); (d) IPG, left kidney at 24 h, grade 5 (severe to very severe injury); (e) KIPG, left kidney at 24 h, grade 4.

Since no lesions were detected in CG or KG, only IPG and KIPG were analyzed regarding differences in the degree of renal injury. No differences were observed between these groups (*p* = 0.63) (Figure 2).

## 4. Discussion

In our study, IP was unable to prevent structural renal tubular damage. Subanesthetic S(+)-ketamine showed no additional beneficial effects for IP but was also not responsible for worsening lesion scores.

The best animal model for IP is controversial. In 2007, using a mouse model, Szwarc et al. were the first to demonstrate that IP could prevent ischemic AKI [11]. In an overview, van den Akker et al. reviewed 13 studies on renal IRI and IP with different* in vivo* models, demonstrating improvements in renal function and histology in most studies. Two studies did not present benefits in renal function in IP: morphological analysis revealed reductions in fibroses, and terminal deoxynucleotidyl transferase dUTP nick end labeling (TUNEL) results were altered [1]. In our study, IP did not protect renal function and histology. A possible explanation is the algorithm used for IP, with longer periods of reocclusion (2 min) and reperfusion (2 min) of the renal artery, as opposed to most of the other studies that used seconds (10, 15, 30, 45, and 60 s) [1]. Besides the fact that the period of initial ischemia was short (30 min), the differences in the cycles of ischemia (2 min) and reperfusion (2 min) in the IP period in our study were longer [3 × 2 min (12 min) compared with 6 × 10 s (1 min) in rats]. This possibility may be supported by the findings of Jiang et al., who studied three different algorithm periods in IP in mongrel dogs, six cycles of 15 s, six cycles of 30 s, and three cycles of 60 s, and reported significant differences among the three algorithms [12]. The effect of the smaller cycle (six cycles of 15 s) was the best. Despite this finding, Serviddio et al. used three algorithms with more prolonged periods of ischemia and reperfusion in IP in rats, 3 cycles of 5 min, 6 cycles of 5 min, and 12 cycles of 5 min, and reported improved kidney function and less necrosis [13]. Unfortunately, the optimal IP algorithm remains unknown. Generally, in smaller animals with faster heartbeats, a shorter period of time can be used (5–10 s) compared with larger animals (30–60 s) for IP because of the correspondingly faster metabolic rate. The duration of ischemia is a determinant for the functional and histological severity of AKI; however, in the literature, we found differences in animal models, the duration of renal ischemia (30 to 90 min), and the time between reperfusion and analysis (24 to 72 h). In this study, we used a short period of ischemia (30 min) because our period of cycles in IP was longer. Our results demonstrated that 30 min is sufficient to produce important renal damage in either morphology or function with significant elevations in serum Cr, BUN, and NGAL. Kidney transplantation is not ordinarily an elective procedure, and ischemic preconditioning cannot always be performed, but IP can be performed because it is a simple intervention. If IP is effective at reducing damage, questions arise, including the best model of IP and the possibility of translating IP results from animal studies to clinical practice in humans. The answers to these questions remain unclear.

In the context of renal transplantation, cold ischemia and the concomitant use of immunosuppressive medications could impair positive strategies for successful reperfusion. McCafferty et al. [14] requested a pause for thought because the potential benefit of IP, as reported in the article by van den Akker et al., may be ineffective at reducing reperfusion injury if the index ischemia has already caused irreversible injury. This possibility may also explain some degrees of lesions not measured in our study.

The N-methyl-D-aspartate receptor (NMDAR) is a heterotetrameric amino acid, part of the glutamate receptor family, originally described in the central nervous system, where it functions as a membrane calcium channel. These receptors definitely participate in various parts of the nephron, including the collecting ducts, glomerulus, and podocytes; therefore, it is not difficult to associate the receptors with a type of renal function regulation. In the neonatal kidney, NMDAR subunits NR3a and NR3b are expressed; Sproul et al. reported the upregulation of NR3a secondary to hypoxia and hypertonicity in a mouse model [15]. NMDARs promote renal vasodilator effects through the nitric oxide pathway [16]. However, NMDAR-mediated activation is associated with enhanced oxidative stress and renal damage, as observed in gentamicin renal injury or hyperhomocysteinemic rats [17, 18]. Antagonism of multiple-target sites of NMDAR seems to show benefits against AKI [7].

Ketamine is a noncompetitive antagonist of the NMDAR that binds at the polyamine-binding site. The protective role of ketamine through NMDAR blockade has been studied in the brain with additional regenerative effects for S(+)-ketamine in cultured neurons [19]. In skeletal muscle, racemic ketamine at a subanesthetic dose has already been described as displaying beneficial effects in IRI [20]. Other NMDAR antagonists have been studied in renal IRI. In 2008, Yang et al. observed improvement in renal dysfunction following ischemia-reperfusion in rats with prior use of an NMDAR antagonist, D-2-amino-5-phosphopentanoic acid (D-AP-5) [21]. In acute tubular necrosis (ATN) promoted by gentamicin, in which overexpression of the NMDAR was observed, another antagonist of the NMDAR, MK-801, also promoted renal protection [18]. Recently Pundir et al. used a rat model of 40 min of bilateral renal ischemia followed by 24 h reperfusion and observed significant protection with ketamine in renal IRI at a significantly higher dose than that used for anesthesia [7]. A subanesthetic dose of S(+)-ketamine had no protective effect on the kidney in this study and did not improve or aggravate the renal damage in IP rats. One argument is that the subanesthetic dose of ketamine that we used may be insufficient to confer protection in this model of renal postconditioning, but the presence of structural lesions in the groups submitted to ischemia may have masked the possible benefits for functional damage. While the effects of NMDAR antagonists on the modulation of glomerular tone are still not fully understood, elevated serum levels of catecholamines, a dose-dependent response to ketamine administration, has a negative role in renal function, supporting our choice of a low-dose regimen with the more potent isomer. The highest scores for tubular lesions have been attributed to the use of S(+)-ketamine in rats associated with higher levels of catecholamines [22]. The use of subanesthetic doses preserves anti-inflammatory activity while reducing sympathomimetic action [23].

Serum creatinine, BUN, and NGAL are widely accepted for assessing renal function and renal histology reveals any parenchymal injuries. Creatinine is the most commonly used biomarker in clinical practice, even though NGAL presents greater sensitivity for the early detection of AKI [1]. Despite the limitations that body weight, mass, and hydration exert on creatinine, our results were consistent with a 24 h ischemic aggression protocol in the groups in which ischemia-reperfusion was performed. In this study, the histological evidence of renal injury corresponded with plasma levels of creatinine and urea. However, functional damage occurred even in the groups not submitted to ischemia (CG and KG), as observed by the increase in NGAL, which was virtually unaltered between *T*0 and *T*2 (KG), but showed similarities with the remaining groups. The increase in NGAL is also considered to be secondary to comorbidities and systemic inflammation, which can be caused by surgical procedures. This biomarker is considered highly sensitive for discriminating AKI, but in this study, its expression was unexpectedly variable and inconclusive [1, 24]. In this work, S(+)-ketamine reduced the functional lesion, as demonstrated by NGAL values, in rats not submitted to the IP protocol. This effect most likely occurred because the drug attenuated inflammation in the right nephrectomy. Unfortunately, in our experimental IP model, we observed structural damage likely secondary to ischemia time, which did not allow the observation of a potential protective effect of the drug in this setting.

Evaluations of the weight of the rats and the control of blood pressure, temperature, and hydration were all adequately performed in our model; the groups exhibited homogeneous behaviors.

The majority of murine studies focus on AKI following bilateral warm ischemia [25]. Evolution to ATN and interstitial inflammatory infiltrate is characteristic in the first 24 h after reperfusion.

The mechanisms involved in pharmacological preconditioning and IP require experimental research that corroborates and improves our current understanding of the intervention models. Reflections concerning the results obtained accrue from trying to determine the optimal algorithm, from control of the intervention and from safe and favorable reproducibility, which permit clear extrapolation to clinical practice.

## 5. Conclusion

In our study, the effect of postconditioning itself was unable to prevent severe structural tubular injury, probably due to the prolonged ischemia time in the algorithm. We conclude that a subanesthetic dose of S(+)-ketamine provided no additional beneficial effects for the postconditioning model, but neither was it responsible for the worst injury scores. The distinction in lesion progression between functional injury and permanent structural damage and how to anticipate and disrupt this complex process remains the subject of future research.

## Figures and Tables

**Table 1:** Descriptive and longitudinal analysis of the biochemical variables within each group.

Collect	*T*0			*T*2			*T*3			Time effect^a^	Contrast analysis
Group	Mean		SD	Mean		SD	Mean		SD		
Cr											
CG	0.430	±	0.142	0.620	±	0.193	0.550	±	0.108	0.021	*T*0 < (*T*2 = *T*3)
KG	0.350	±	0.053	0.520	±	0.079	0.570	±	0.082	0.0001	*T*0 < (*T*2 = *T*3)
IPG	0.390	±	0.074	0.820	±	0.274	2.60	±	1.67	0.0001	*T*0 < *T*2 < *T*3
KIPG	0.418	±	0.117	0.964	±	0.112	3.26	±	1.83	0.0001	*T*0 < *T*2 < *T*3
NGAL											
CG	5.8	±	4.9	20.0	±	23.0	217.2	±	65.4	0.0001	*T*0 < *T*2 < *T*3
KG	5.3	±	4.5	5.2	±	2.7	226.5	±	96.6	0.0001	(*T*0 = *T*2) < *T*3
IPG	10.1	±	10.4	37.7	±	41.3	343.6	±	50.6	0.0001	*T*0 < *T*2 < *T*3
KIPG	20.8	±	19.9	66.0	±	104.9	374.0	±	17.7	0.0001	*T*0 < *T*2 < *T*3
Na^+^											
CG	132.4	±	3.2	130.9	±	4.1	134.4	±	2.2	0.091	
KG	130.1	±	4.0	132.9	±	1.7	133.8	±	3.3	0.025	*T*0 < (*T*2 = *T*3)
IPG	128.5	±	2.8	128.2	±	2.4	128.6	±	1.9	0.90	
KIPG	130.7	±	3.3	129.4	±	2.7	131.4	±	3.6	0.32	
BUN											
CG	55.0	±	14.8	63.7	±	16.3	56.3	±	11.6	0.17	
KG	50.6	±	3.1	58.0	±	3.9	60.1	±	8.5	0.004	*T*0 < (*T*2 = *T*3)
IPG	45.4	±	5.3	60.8	±	7.2	174.5	±	70.1	0.0001	*T*0 < *T*2 < *T*3
KIPG	46.4	±	5.9	62.9	±	4.2	206.0	±	76.2	0.0001	*T*0 < *T*2 < *T*3

SD: standard deviation; Cr: creatinine; NGAL: neutrophil gelatinase-associated lipocalin; Na^+^: sodium; BUN: blood urea nitrogen; CG: control group; KG: subanesthetic S(+)-ketamine continuous infusion group; IPG: ischemic postconditioning (IP) group; KIPG: subanesthetic S(+)-ketamine continuous infusion + IP group.

^a^ANOVA for repeated measures within each experimental group.

**Table 2:** Repeated measures ANOVA one factor and contrast analysis among the groups.

	Main effect		Interaction	Contrast analysis among groups		
	Group	Time		Time point match	*p* value	Commentaries
Cr	0.0001	0.0001	0.0001	*T*0 × *T*2	0.034	(CG = KG) < KIPG, IPG
				*T*0 × *T*3	0.0001	(CG = KG) < (IPG = KIPG)
				*T*2 × *T*3	0.0001	(CG = KG) < (IPG = KIPG)

NGAL	0.0008	0.0001	0.081	*T*0 × *T*2	0.041	KG < (CG = IPG = KIPG)
				*T*0 × *T*3	0.54	CG = KG = IPG = KIPG
				*T*2 × *T*3	0.11	CG = KG = IPG = KIPG

Na	0.0001	0.014	0.13	*T*0 × *T*2	0.096	CG = KG = IPG = KIPG
				*T*0 × *T*3	0.21	CG = KG = IPG = KIPG
				*T*2 × *T*3	0.30	CG = KG = IPG = KIPG

Ur	0.0001	0.0001	0.0001	*T*0 × *T*2	0.004	(CG = KG) < (IPG = KIPG)
				*T*0 × *T*3	0.0001	(CG = KG) < (IPG = KIPG)
				*T*2 × *T*3	0.0001	(CG = KG) < (IPG = KIPG)

Cr: creatinine; NGAL: neutrophil gelatinase-associated lipocalin; Na^+^: sodium; BUN: blood urea nitrogen; CG: control group; KG: subanesthetic S(+)-ketamine continuous infusion; IPG: ischemic postconditioning (IP) group; KIPG: subanesthetic S(+)-ketamine continuous infusion + IP group.

## Abbreviations

AKI: Acute kidney injury
ANOVA: Analysis of variance
ATN: Acute tubular necrosis
BUN: Blood urea nitrogen
CG: Control group
FDA: Food and Drug Administration
IP: Ischemic postconditioning
IPG: Ischemic postconditioning group
IRI: Ischemia-reperfusion injury
KG: Subanesthetic S(+)-ketamine group
KIPG: Subanesthetic S(+)-ketamine ischemic postconditioning group
LCA: Left carotid artery
*T*0: 15 minutes after the beginning of the anesthesia
*T*1: After 30 min of left renal artery clamping
*T*2: After 30 min of reperfusion and cycles of ischemic postconditioning
*T*3: After 24 h of reperfusion
NGAL: Neutrophil gelatinase-associated lipocalin
NMDA: N-Methyl-D-aspartate
NMDAR: N-Methyl-D-aspartate receptor
RIJV: Right internal jugular vein
RL: Ringer lactate
ROS: Reactive oxygen species.
